# Correlation between sensory attributes and Metabolomic profiles of cocoa liquor from different cacao genotypes

**DOI:** 10.1016/j.fochx.2025.102498

**Published:** 2025-04-25

**Authors:** Enik Nurlaili Afifah, Indah Anita Sari, Agung Wahyu Susilo, Hendy Firmanto, Abdul Malik, Eiichiro Fukusaki, Sastia Prama Putri

**Affiliations:** a Department of Biotechnology, Graduate School of Engineering, Osaka University, 2-1 Yamadaoka, Suita, Osaka 565-0871, Japan; b Department of Agronomy, Faculty of Agriculture, Universitas Gadjah Mada, Jl. Flora, Bulaksumur, Sleman district, Daerah Istimewa Yogyakarta 55281, Indonesia; c Indonesian Coffee and Cocoa Research Institute, Jl. PB. Sudirman 90, Jember, Jawa Timur 68118, Indonesia; d Industrial Biotechnology Initiative Division, Institute for Open and Transdisciplinary Research Initiatives, 2-1 Yamadaoka, Suita, Osaka 565-0871, Japan; e Osaka University Shimadzu Omics Innovation Research Laboratories, International Center for Biotechnology, Osaka University 2-1, Yamadaoka, Suita, Osaka, Japan

**Keywords:** Cocoa liquor, Flavor, Genotype, Metabolomics, Quality, Sensory analysis, *theobroma cacao*

## Abstract

Flavor is a critical quality of cacao beans and is significantly influenced by the genotype. Relying solely on sensory evaluation to describe the complex flavors of different cacao genotypes is insufficient because of the reliance on human expertise and the need for robust validation. This study aimed to enhance the understanding of flavor quality in different cacao genotypes by investigating the correlation between sensory attributes and metabolite profiles using metabolomics-based approach and sensory analysis. Cacao genotypes can be categorized based on their sensory attributes and metabolite profiles. The ICCRI09-genotype exhibited outstanding flavors, which were significantly associated with non-volatile and volatile compounds (e.g. Organic acids, some sugars, 3-methylbutanal, 2,3-butanediol, benzaldehyde, linalool, trans-linalool oxide, isobutyl acetate, etc.) (*P* < 0.05). In contrast, theobromine, epicatechin, and catechin, which are linked to bitterness and astringency, were abundant in KW516-Forastero. These findings have significant implications and provide a scientific basis for authenticating the high flavor quality of cacao.

## Highlights

• Flavor quality of cocoa liquor correlates with sensory attributes and metabolomic profiles.
• ICCRI 09 exhibits distinct sensory and metabolomic traits compared to the original Trinitario and Forastero.
• Both volatile and nonvolatile components contribute to cacao flavor complexity.

## 1 Introduction

Cacao (*Theobroma cacao*), a major cash crop in Ghana, Indonesia, and Brazil (Tennhardt et al., 2023), has significant economic value as the primary ingredient in chocolate, a global industry worth billions of dollars (Tuenter et al., 2020). Beans provide the fundamental flavor profile of chocolate, which includes bitterness, sweetness, acidity, and astringency, and notable notes of fruitiness, nuttiness, floral, and spiciness, creating a rich, layered taste (Zapata-Alvarez et al., 2024).

The flavor of cocoa is ultimately determined by the chemical composition of the beans, which varies across genotypes (Kadow et al., 2013)(Toker et al., 2020). The levels of storage polysaccharides, proteins, and polyphenols present in beans determine the types and amounts of precursors formed during fermentation, drying, and roasting. These precursors play crucial roles in flavor development and significantly influence the final flavor profile(Kongor et al., 2016).

Several factors influence the flavor profile of cocoa, and the variety of cacao plays a significant role(Colonges, Jimenez, et al., 2022). Each variety has distinct flavor characteristics(Sukha et al., 2017)(Santander Muñoz et al., 2020). For instance, Criollo and some Trinitario varieties offer a balance between complexity and robustness, often featuring fruity, floral, and nutty notes along with a rich cocoa base(Castro-Alayo et al., 2019). In contrast, Forastero beans are generally less complex and bitter than Criollo (Motamayor et al., 2008) beans(Colonges, Loor Solorzano, et al., 2022). Among these varieties, Trinitario is the most predominant in cacao plantations worldwide, owing to its vigorous growth and moderate resistance to pests and diseases. However, Trinitario and its hybrid progenies exhibited diverse flavor characteristics resulting from genetic segregation, underscoring the importance of evaluating flavor quality(Kongor et al., 2016)(Febrianto & Zhu, 2019).

High-flavor-quality beans are often assessed by the color of the bean cotyledons in the Criollo and Forastero varieties. However, according to Afifah et al.(Afifah et al., 2024), this approach is challenging to apply to hybrid progenies because of the variability in fresh bean color. Recently, sensory evaluation has become a critical method for understanding the flavor characteristics of cacao samples(D. N. Putri et al., 2024). However, sensory evaluation alone is insufficient to fully understand the complex flavor profile of cocoa. This method relies heavily on human skill and expertise to capture flavor attributes, leading to challenges in achieving consistent conclusions regarding cocoa flavor quality. Therefore, it is necessary to validate sensory results using more robust methods.

Metabolomic analysis provides valuable insights for the comprehensive profiling of the chemical composition of cacao beans (Herrera-Rocha et al., 2024) and other agricultural products (Balcázar-Zumaeta et al., 2023) (Shen et al., 2023). This approach has been widely used to identify food quality markers by correlating chemical profiles with sensory attributes (Zhang et al., 2024) (Kim et al., 2023). Gas chromatography-mass spectrometry (GC–MS) has been increasingly employed for chemical analyses of cacao because of its reproducibility, stability, and ease of use (S. P. Putri et al., 2022). Combined with sensory analysis, this method can significantly enhance our understanding of the flavor characteristics of different cacao genotypes. Correlating the metabolomic profiles of cocoa with sensory attributes can validate sensory perceptions and provide a scientific basis for understanding flavor characteristics by identifying the specific compounds responsible for certain attributes(Y. Zhao et al., 2024)(L. Zhao et al., 2023)(Kim et al., 2023).

Previous studies on the flavor profiles of different cacao genotypes have often focused solely on metabolomic analysis to identify volatile and non-volatile compounds in unfermented and fermented cacao samples, without incorporating sensory evaluation (Velásquez-Reyes et al., 2023) (Qin et al., 2017). Conversely, some studies have evaluated only the sensory profiles of cacao genotypes without conducting metabolite profiling (Zapata-Alvarez et al., 2024) (I. A. Sari et al., 2022). The relationship between the overall metabolite profiles (both volatile and non-volatile) and sensory attributes of cacao genotypes remains complex and not fully understood. This study aimed to address this gap by correlating metabolite profiles with sensory evaluations to better understand the flavor characteristics of different cacao genotypes. Additionally, based on the recent findings of Afifah et al.(Afifah et al., 2024), who showed that hybrid progeny from a cross between the Trinitario variety has promising flavor potential, this study comprehensively examined the flavor components of these hybrids by correlating their metabolite profiles with sensory attributes.

Understanding the relationship between the overall chemical composition and complex sensory attributes of cocoa liquor from different genotypes is crucial for the accurate characterization of the driving innovation in cocoa breeding and the prediction of flavor profiles in cocoa. Furthermore, identifying and understanding the compounds related to sensory attributes is essential for ensuring product quality(D. N. Putri et al., 2024), aligning with consumer preferences (Sato et al., 2021), and responding to market trends (A. B. T. Sari et al., 2023). The goal of this study was to identify the correlation between the sensory attributes and chemical profiles of different cacao genotypes using a combined sensory evaluation and metabolomic approach.

## 2 Materials and methods

### 2.1 Cacao sample

Cacao genotypes from ICCRI (Indonesian Coffee and Cocoa Research Institute), namely “KW 516” (Forastero variety), “SULAWESI 01” (Trinitario variety), and “ICCRI 09” (a hybrid from Trinitario), are known for their good productivity, resistance, and distinct flavor characteristics. Therefore, these samples were used in this study. These genotypes were propagated vegetatively, resulting in clones. These clones were grown in Jember, East Java Province, Indonesia, under the following climatic conditions: an altitude of 45 m above sea level, an average temperature of 28 °C, and an average rainfall of 300 mm in 2024. The cacao clones underwent similar cultivation management by the Indonesian Coffee and Cocoa Research Institute (ICCRI), with plants reaching a height of approximately 3 m at 5 years of maturity. Cacao pods were harvested during the rainy season in April 2024, shelled, and pooled into a fermentation box for the fermentation process (Fig. S1).

### 2.2 Post-harvest processing

All samples underwent similar postharvest processing. After breaking the pods, the cacao samples were pooled into fermentation boxes as shown in Fig. S2 with a capacity of 10 kg per clone. Fermentation was conducted at an environmental temperature of 28 °C and 85 % humidity for 114 h. According to the ICCRI fermentation standard, the beans were manually turned off after 48 h to ensure homogeneous fermentation. The fermentation endpoint was determined using a cutoff test. After fermentation, beans from each clone were spread on a square bamboo tray (Fig. S1) and naturally dried under sunlight for four days. Each day, the beans were manually mixed to achieve a moisture content of 7–8 %. After drying, the beans were subjected to fermentation index measurements and cut test analyses.

The procedure for roasting and producing cocoa liquor was based on the ISCQF (International Standards for the Assessment of Cocoa Quality and Flavor) protocol (Gutiérrez, 2025) and the ICCRI standard, as follows: After drying, the fermented beans of each genotype were placed separately on perforated metal trays. The samples were roasted in an oven at 120 °C for 30 min, following a preheating period of 15 min at the same temperature. The actual roasting temperature and time for each clone were adjusted based on the moisture content of the beans and their size (weight of 100 grains). The timing was measured from 2 °C below the set point. After roasting, the beans were cooled immediately to stop roasting. To prepare the cocoa liquor, 500 g of roasted beans was winnowed to separate the nibs from their shells (using a basic winnower, Cocoa TownTM, Alpharetta, USA). The shelled cocoa nibs were ground using a melanger until smooth cocoa liquor was obtained. All samples were then packed, vacuum-sealed, and stored in a fridge at −30 °C until further analysis.

### 2.3 Degree of fermentation index and cut test analysis

Dried cacao beans from each clone were collected (100 grains per sample) and used for the cutting test and fermentation index analysis. For the cut test analysis, beans were manually cut lengthwise into two parts to expose the cotyledons. Each sample was placed on a white background, analyzed, and classified based on the color and texture of the exposed surface, as shown in Fig. S2.

The fermentation index was measured according to a previous study by Kongor et al. (Edem Kongor et al., 2013) using the Gourieva and Tsernetivinov methods. Five dried beans were ground using a mortar and pestle, and then approximately 0.5 g of the crushed beans were extracted in 50 mL of a mixed solution (composed of methanol and HCl in a 97:3 *v*/v ratio). The sample was then left to homogenize in a refrigerator (8 °C) for approximately 20 h. The solution was filtered using Whatman paper (No.1) and the filtered solution was analyzed using a Shimadzu UV-1601 UV–vis spectrophotometer in the wavelength range of 400–700 nm. The fermentation index was calculated based on the ratio of the absorbance values at 460 nm and 530 nm.

### 2.4 Sensory evaluation

The cocoa liquor of each clone was tested by six trained panelists from the Indonesian Coffee and Cocoa Research Institute (ICCRI) board members, including four females and two males (30–50 years old). Cocoa samples for sensory analysis were prepared according to (The International Standards for the Assessment of Cocoa Quality and Flavor (ISCQF) protocol (Gutiérrez, 2025). Approximately 1–2 g of cocoa liquor from each clone were put in a cup then heated at 48–50 °C until cocoa paste was melted. Then, a melt cocoa liquor was served to the panelists. All samples were maintained blindly using alphabet codes and evaluated in random order for all blind evaluations. Twenty-one attributes according to the ISCQF protocol (cocoa, acidity, astringency, fresh fruit, browned fruit, floral, vegetal, woody, spicy, nutty, sweet, browned roast, dusty, meaty, putrid, smoky, moldy, other off-flavors, global quality, overall flavor, and uniqueness) were scored ranging from zero to ten with the following criteria: 0 (no attribute detected), 1 (just a trace), 2 (low intensity), 3–5 (clearly characterized), 6–8 (dominant in the sample), 9–10 (Strong intensity).

### 2.5 Sample preparation and GC–MS non-volatile analysis

The GC–MS non-volatile analysis was performed based on a previous study(Hanifah et al., 2022). The cocoa liquor (5 g) was crushed in a polycarbonate tube using a stainless-steel ball. The tube was immersed in liquid nitrogen and crushed into powder using a multibead shocker (ST-5010 PCR; Yasui Kikai, Osaka, Japan). Approximately 5 mg of cocoa powder from each sample was prepared in 2 mL microtubes, with three replicates for each sample. Each sample was then mixed with a solvent composed of methanol (Wako Chemical, Osaka, Japan), chloroform (Kishida Chemical, Osaka, Japan), and ultrapure water (Wako Chemical) in a ratio of 2.5:1:1 *v*/v/v, containing 0.1 mg/mL ribitol as an internal standard. Blank samples (containing only the mixed solvent) were also prepared in 2 mL tubes. The solutions were vortexed to homogenize the samples and then incubated in a shaker at 1200 rpm and 37 °C for 30 min. The supernatant was obtained by centrifugation at 4 °C and 10,000 ×*g* for 3 min. Subsequently, 600 μL of the supernatant was transferred to a 1.5 mL tube, and 300 μL of ultrapure water was added. This mixture was vortexed and centrifuged again for 3 min at 4 °C and 10,000 ×*g*. Then, 200 μL of the aqueous phase was transferred to a new 1.5 mL tube and sealed with a holed cap. Additionally, 200 μL of the aqueous phase from each sample was pooled to create quality control (QC) samples, which were also transferred to a new 1.5 mL tube and sealed with a holed cap. The samples were then evaporated for 2 h at room temperature using a centrifuge concentrator.

Oximization and silylation were performed for derivatization. First, 100 μL of methoxyamine hydrochloride (20 mg/mL in pyridine; Sigma-Aldrich, St. Louis, MO, USA) was added to the samples, and the mixture was incubated in a thermomixer for 90 min at 30 °C and 1200 rpm. Next, 50 μL of *N*-methyl-*N*-trimethylsilyl-trifluoroacetamide (GL Sciences, Inc., Tokyo, Japan) was added to the sample mixture and incubated again for 30 min at 37 °C and 1200 rpm. Finally, 100 μL of each sample was transferred to a vial for GC–MS analysis.

A GC–MS QP2010 Ultra system (Shimadzu, Kyoto, Japan) was used for the analysis. The system was equipped with a Proguard 5 MS/NP column (GL Sciences, 0.25 μm) and an autosampler (AOC-20i/s; Shimadzu). Before the samples were analyzed, tuning and calibration checks were performed using mass spectrometry. Once tuning and calibration were confirmed, the samples were injected at a split ratio of 25:1 (*v*/v) at an injection temperature of 270 °C and analyzed in random order. Hydrogen was used as the carrier gas, with a linear velocity of 39.0 cm/s and a flow rate of 1.2 mL/min was used in this analysis. The column temperature was initially set at 80 °C for 4 min, then increased by 15 °C per minute to 330 °C, and maintained at 330 °C for 8 min. Ions were generated using the electron ionization (EI) method with a filament bias voltage of 70.0 V. EI mass spectra were recorded over a mass range of 85–500 *m*/*z* with an event time of 0.15 s. At the beginning of the analysis, a standard alkane mixture (C10–C40) was injected to determine retention indices (RIs) for tentative identification.

### 2.6 Volatile compounds analysis by HS-SPME arrow GC–MS

The volatile components of cocoa liquor from different cacao genotypes were extracted using the HS HS-SPME arrow and analyzed by GC–MS, following previous studies by Velásquez-Reyes et al.(Velásquez-Reyes et al., 2023) with modification. A solid form of cocoa liquor was broken into pieces, and approximately 2, 5 g of each sample was weighed using three replicates. QC sample was also prepared by weighing 2,5 g from each sample and mixed to obtain a QC pool sample (*n* = 3). All samples were then placed in a 20 mL screw vial and closed with a magnetic screw cap. The SPME procedure was conducted automatically using a multifunctional autosampler (AOC-6000 by Shimadzu) fitted with a DVB/CAR/PDMS (Divinylbenzene/Carboxen/Polydimethylsiloxane) fiber (20 mm × o.d. 1.1 mm, df = 100 μm; Shimadzu). Prior to sample extraction, the fibers were preconditioned at 250 °C for 15 min. The samples were incubated at 60 °C for 30 min with agitation. Extraction was carried out at 60 °C and shaken at 250 rpm for 30 min. Following extraction, the fiber was placed into the GC inlet, and the analyte was injected via thermal desorption at 250 °C for 2 min.

A gas chromatograph–mass spectrometer (GCMS-TQ8050 NX; Shimadzu) equipped with a capillary GC column (InertCap FFAP, 0.32 mm × 60 m, 0.50 μm; GL Sciences) was utilized in this analysis. The analyte desorbed from the SPME fibers was injected at a split ratio of 25:1 (*v*/v). A helium carrier gas with a linear velocity of 40.7 cm/s. The column oven temperature was initially set at 40 °C for 5 min, then gradually increased to 250 °C at a rate of 3 °C/min, and maintained at 250 °C for 45 min. The MS was operated in scan mode (*m*/*z* 24–350). Both the ion source and interface temperatures were set at 250 °*C. prior* to the analysis, a standard of fatty acid ethyl esters (FAEEs, C4–C24) was injected to calculate the RIs for tentative identification.

### 2.7 Raw data processing

Raw data processing was carried out by converting the GC–MS data into an AIA file format (AIA) using the GC–MS Solution software (Shimadzu). The AIA files were then converted into. Abf format (analysis-based file) using the ABF Converter program (available for download from website). Baseline correction, peak filtering, alignment, noise reduction, and peak annotation were performed using MS-DIAL version 4.4 (RIKEN, Kanagawa, Japan), an open-source software freely available for download from the official MS-DIAL website. The analysis was based on the retention index (RI) and mass spectral information from the GL-Science DB spectral database, which is also available for download from the same site. Tentative annotations were confirmed by comparing the detected peaks with the data from the NIST library (NIST/EPA/NIH EI-MS Library) included in the GC/MS Solution software packages. Metabolites with a similarity score greater than 80 % (from annotations using both the MS-DIAL and NIST libraries) were included in the analysis. The annotated metabolites were normalized to an internal standard. For the HS-SPME arrow GC/MS analysis, the intensities were normalized using the locally weighted regression scatterplot smoother (LOWESS) method. Filtering was performed by removing metabolites with relative standard deviation (RSD) values greater than 30 % from the QC samples.

### 2.8 Statistical analysis

All the data obtained from this study including metabolite, sensory attribute, fermentation index, and cut test analysis data were analyzed using ANOVA (analysis of variance) to observe the significant variation within the sample, then followed by a post-hoc Tukey's honest significant difference (HSD) test at α = 5 %. Analysis of variance, biplot analysis, and Pearson's correlation were performed using the R software version 4.3.2 (2023−10−31), an open-source statistical computing environment. Principal component analysis (PCA) and partial least squares (PLS) regression analyses were performed using SIMCA-P+ version 13 (Umetricts, Umea, Sweden), a commercial software for multivariate data analysis. PCA was used to visualize metabolite data within the cacao genotypes. PLS regression analysis was conducted to determine the relationships between metabolite profiles as explanatory variables (x) and sensory attributes as response variables (y) (Kim et al., 2023) for different cacao genotypes. The PLS regression model was then validated by cross-validation using the R^2^ (a coefficient of determination which represents the proportion of the variance in the dependent variable) and Q^2^ (model's predictive ability) parameters obtained from a random test with *n* = 200.

## 3 Results and discussion

### 3.1 Sensory characteristics of cocoa genotypes

**Fig. 1:**
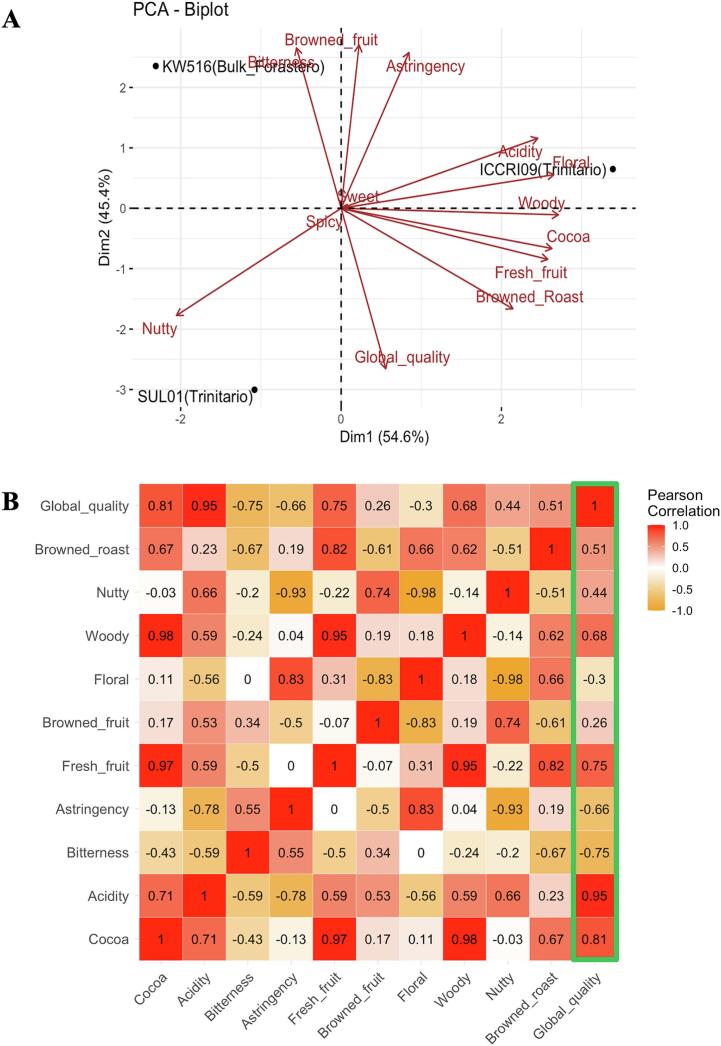
Biplot analysis showing the sensory characteristics of cacao genotypes (A). Heatmap depicting the relationships between sensory attributes in cacao genotype based on Pearson's correlations (B).

The sensory evaluation results of the different cacao genotypes were statistically visualized using a PCA biplot, as shown in Fig. 1A. The first two principal components, PC1 and PC2, explained 54.6 % and 45.4 % of the variance, respectively, effectively differentiating the cacao genotypes. According to the ISCQF protocol(Gutiérrez, n.d.), experts advise that the sensory attributes of cacao can be categorized into core attributes, complementary attributes (which may or may not be perceived), and off-flavors. In this study, 21 sensory attributes based on ISCQF standards were used to examine the flavor quality of various cacao genotypes. Of these, 11 attributes were identified in the cacao genotype, including bitterness, astringency, acidity, and cocoa (core tastes), as well as browned roast, browned fruit, floral, woody, fresh fruit, and nutty (complementary attributes), and global quality. Therefore, this discussion focuses on these eleven attributes. The biplot shows that no off-flavors were detected in any of the genotypes, indicating that all samples underwent an optimal fermentation process. This result is supported by the degree of fermentation index and cut test analysis, as presented in Fig. S2.

Cacao genotype “ICCRI 09” was dominated by outstanding sensory notes such as floral, woody, cocoa, fresh fruit, browned roast, and global quality. In addition, the Trinitario variety SULAWESI 01 was distinguished by its nutty flavor, although it exhibited only simple flavor attributes. In contrast, the Forastero variety “KW 516” was dominated by the basic flavors of bitterness and astringency, along with the complementary attribute of browned fruit. Interestingly, although both ICCRI 09 and SULAWESI 01 are Trinitario varieties, their flavor profiles differed significantly. ICCRI 09 is a cross between two Trinitario varieties, whereas SULAWESI 01 is a hybrid of the Criollo and Forastero varieties. Previous studies have qualitatively reported that breeding cacao between the Trinitario and Forastero varieties can yield promising results, enhancing both resistance and flavor quality study (Colonges, Jimenez, et al., 2022). However, this study quantitatively revealed that the flavor quality of the ICCRI 09 variety was better than that of the original Trinitario variety. In contrast, KW 516, a Forastero variety, exhibits basic or simple flavors. A previous study (Colonges, Jimenez, et al., 2022) noted that the Forastero variety predominantly produces basic or simple flavor attributes. In contrast, the Trinitario hybrid variety demonstrated genetic and aromatic segregation, which led to greater flavor variability.

To further clarify the sensory attributes of the different cacao genotypes, a Pearson's correlation heatmap was generated (Fig. 1B). The results indicated that the global quality of cocoa liquor from different genotypes was positively correlated with the attributes of cocoa (0.81), acidity (0.95), fresh fruit (0.75), and wood (0.68). “Global quality” refers to the overall cocoa quality. These findings suggest that cocoa, acidity, fresh fruit, and woody notes contribute significantly to the overall quality. Notably, ICCRI 09 exhibited higher global quality, suggesting that this clone may have potential as a high-flavor cocoa preferred by chocolate consumers. Previous studies have found that cocoa samples with higher astringency levels tend to have a lower flavor and overall acceptability (Palma-Morales et al., 2024). Therefore, flavor is a critical factor in consumer acceptance and preference, and high-flavor cocoa commands a higher market price.

### 3.2 Metabolite profile of different cacao genotype

**Fig. 2:**
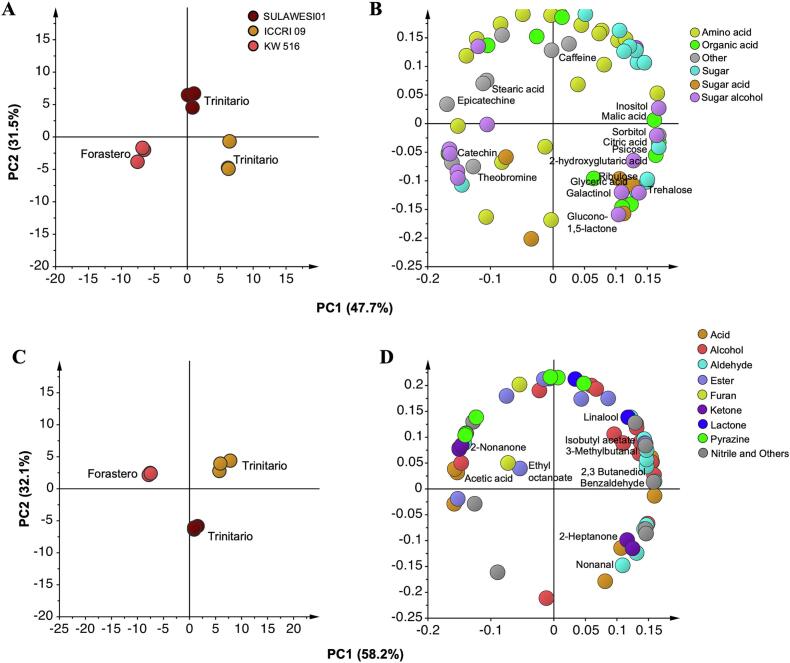
The PCA score plots (A) and loading plots (B) for non-volatile metabolites, along with the score plot (C) and loading plot (D) for volatile metabolites, were generated from GC–MS-based metabolomic analysis using auto-scaling and no transformation, with three replicates. In the score plots, circles represent cacao clones, whereas in the loading plots, circle indicate metabolites contributing to the observed separation. Different colors correspond to various metabolite classes, and highlighted metabolite names denote the key compounds driving sample clustering.

In total, 70 non-volatile metabolites were detected, including amino acids, organic acids, sugars, and other compound classes (Table S1). Additionally, HS-SPME Arrow GC/MS analysis identified 66 volatile metabolites comprising compound classes, such as acids, alcohols, esters, ketones, aldehydes, furans, lactones, and nitriles (Table S2). Statistical analyses were conducted using PCA, as shown in Fig. 2. The nonvolatile metabolite profiles revealed distinct differences between genotypes (Fig. 2A). PC1, which explained 50.8 % of the variance, clearly distinguished ICCRI 09, SULAWESI 01, and KW 516, and was aligned with the sensory evaluation PCA biplot. The loading plot (Fig. 2B) highlighted that the higher-flavor genotype ICCRI 09 exhibited a greater presence of organic acids and sugars.

**Fig. 3:**
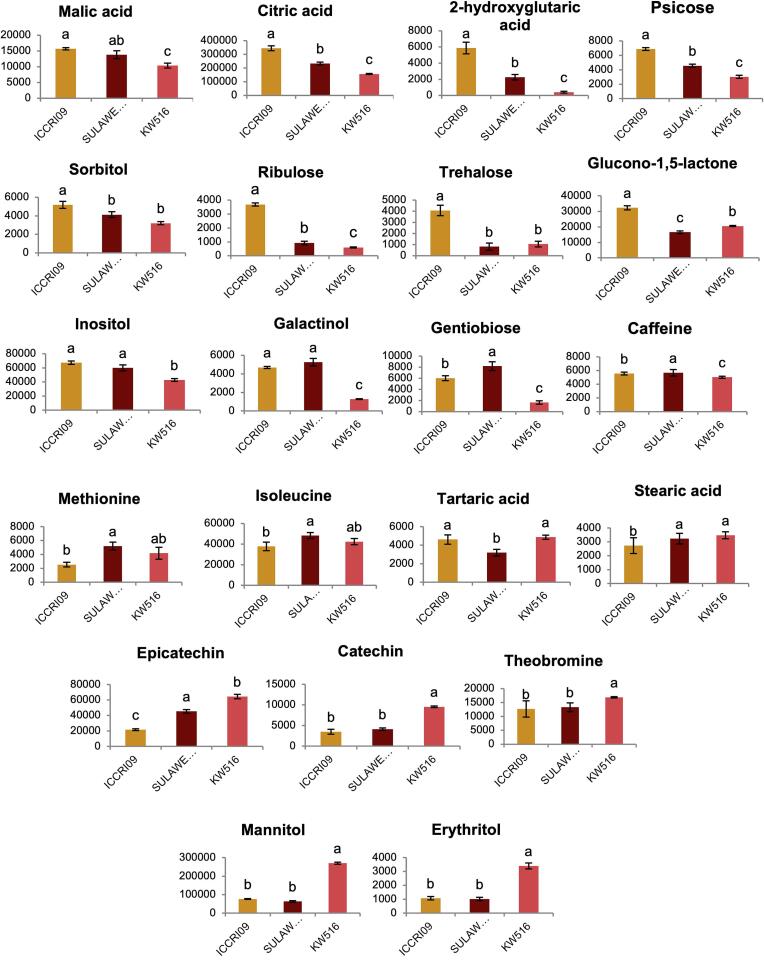
The bar graphs display the relative intensity of the top VIP non-volatile metabolites highlighted in the loading plot, which are highly correlated with the sensory attributes shown in the PLS plot. The vertical axis represents the relative intensity, whereas the horizontal axis indicates the cacao clones. Bars labeled with the same letters do not show a statistically significant difference according to Tukey's HSD test, with a *p-value* < 0.05.

The relative intensities of these highlighted metabolites, presented in Fig. 3, showed that compared to Forastero (KW 516) and Trinitario (SULAWESI 01), the ICCRI 09 clone, which had more complex sensory attributes, exhibited significantly higher levels of organic acids such as citric acid, malic acid, and 2-hydroxyglutaric acid. Additionally, metabolites from sugar and derivative groups, such as glucono 1,5-lactone, trehalose, sorbitol, inositol, psicose, ribulose, and galactinol, were found at higher levels. Previous studies have highlighted that the cocoa flavor is composed of a mixture of hundreds of compounds, including organic acids such as citric acid and malic acid, which contribute to acidity(Kim et al., 2023). Citric acid and succinic acid were particularly dominant in the cocoa liquor of Criollo types(Velásquez-Reyes et al., 2023), which are known for their sensory attributes such as sweet lemon, red berries, and tropical fruit acidity(Papalexandratou et al., 2019). Organic compounds are crucial for balancing the cocoa flavor(Holm et al., 1993). Moreover, reactions between sugars and amino acids can produce volatile aromas in roasted foods and cocoa(Scalone et al., 2019). Previous studies have primarily focused on nonvolatile compounds, such as caffeine and theobromine, and their ratios to differentiate Forastero and Criollo genotypes(Velásquez-Reyes et al., 2023)(Zapata-Alvarez et al., 2024). However, this study reports the non-volatile organic acids and sugars that are abundant in a hybrid of cacao with outstanding sensory characteristics.

The Trinitario, SULAWESI 01, and ICCRI 09 groups showed significantly higher galactinol, malic acid, and inositol levels than the Forastero group. These sugars may enhance the flavor attributes of the two genotypes. Interestingly, although both genotypes were from the Trinitario variety, ICCRI 09 had more complex sensory attributes that aligned with higher levels of nonvolatile organic acids and sugars. Additionally, non-volatile metabolites, such as gentiobiose, isoleucine, and methionine, were significantly more abundant in SULAWESI 01. Previous studies have reported that sugars and amino acids are precursors of flavor development(Qin et al., 2017). These compounds were found at high concentrations in cacao during fermentation(Balcázar-Zumaeta et al., 2024). The correlation between these compounds and the sensory attributes is further discussed in the correlation analysis. Furthermore, KW 516 exhibited significantly higher levels of alkaloids and polyphenols, such as theobromine, epicatechin, catechin, and stearic acid. Polyphenol content has an inverse relationship with flavor quality in cocoa and is typically higher in the Forastero type(Febrianto & Zhu, 2019)(Vázquez-Ovando et al., 2016).

**Fig. 4:**
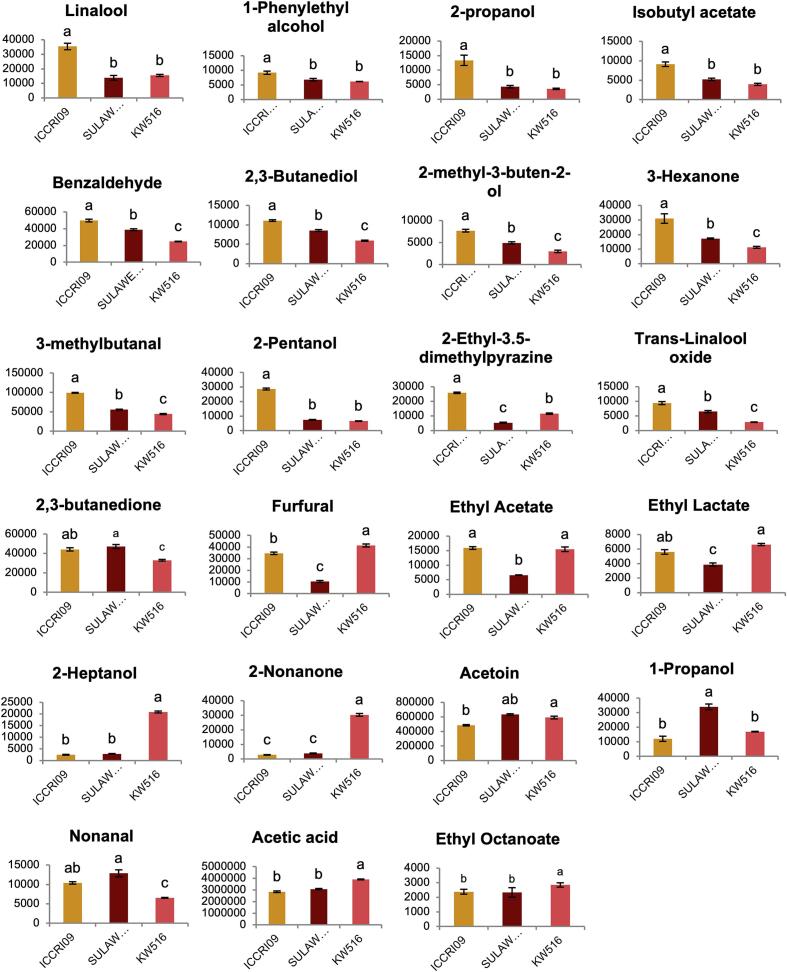
The bar graphs represent the relative intensity of the top VIP volatile components highlighted in the loading plot, which are highly correlated with the sensory attributes shown in the PLS plot. The vertical axis represents the relative intensity, whereas the horizontal axis indicates the cacao clones. Bars labeled with the same letters do not show a statistically significant difference according to Tukey's HSD test, with a *p-value* < 0.05.

PCA of the volatile components, as shown in Fig. 2C, were consistently revealed a clear separation between the cacao genotypes. According to the loading score (Fig. 2D) and bar graph (Fig. 4), ICCRI 09 was notably characterized by an abundance of volatile alcohols, aldehydes, pyrazines, and esters such as 2,3-butanediol, linalool, isobutyl acetate, 3-methylbutanal, 2-Ethyl-3.5-dimethylpyrazine, and benzaldehyde. SULAWESI 01 showed higher levels of 2-heptanone and nonanal compounds. In contrast, acetic acid, ethyl octanoate, and 2-nonanone were predominant in Forastero KW 516. Previous studies have reported that Criollo type cocoa liquor (the finest cocoa) is characterized by benzaldehyde and 3-methylbutanal, whereas Forastero has higher levels of acetic acid and ethyl octanoate. This study reports the key components in a hybrid of Trinitario genotypes with outstanding sensory profiles. Benzaldehyde is associated with cherry, almond, and fruity aromas, while 3-methylbutanal is associated with malted and cocoa aromas(Colonges, Jimenez, et al., 2022). Acetic acid contributes to the flavor of sour vinegar. The association between the sensory profiles and volatile components of these genotypes is discussed in more detail in the correlation analysis.

### 3.3 The correlation between sensory attributes and metabolite profiles of cacao genotypes

**Fig. 5:**
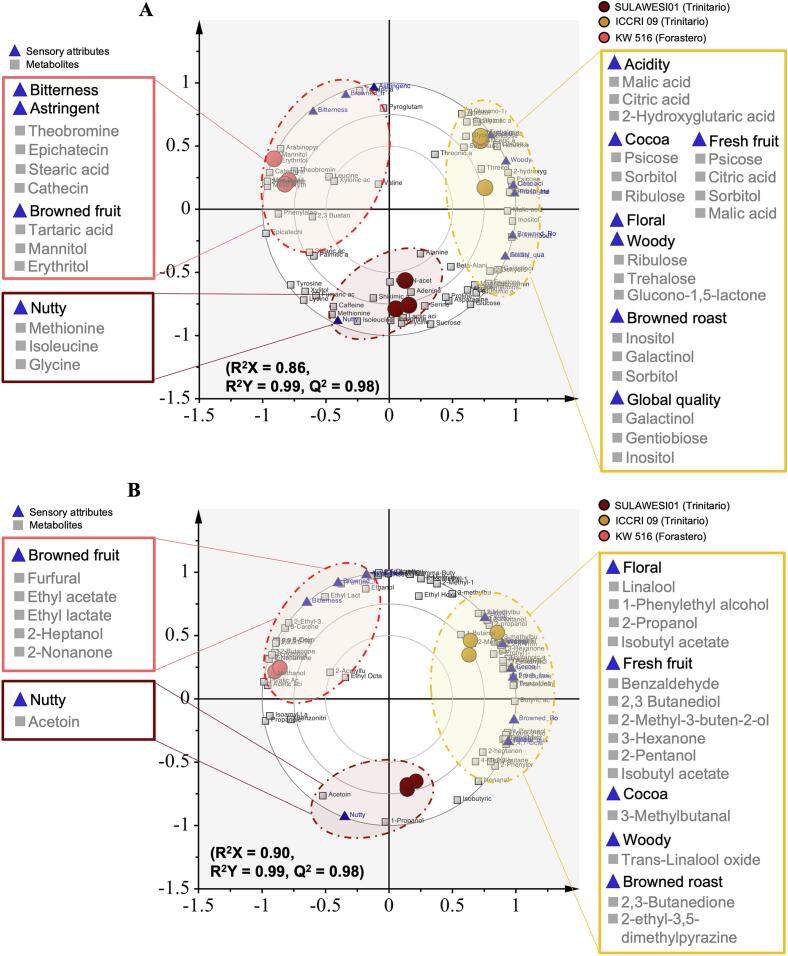
PLS regression plot illustrating the relationships between sensory attributes and metabolite profiles of cacao genotypes: non-volatile compounds (A) and volatile compounds (B).

The relationship between the sensory properties and metabolite profiles of cocoa liquors from different genotypes was analyzed using PLS regression (Fig. 5A and 5 B). As shown in Fig. 5A, the model demonstrates the contribution of non-volatile metabolites (x, *n* = 70) as explanatory variables for sensory attributes (y, *n* = 11). Because nonvolatile metabolites influence the balance of taste and aroma properties in cocoa(Holm et al., 1993), this model was constructed using all the sensory profiles detected in this study to explore the relationship between nonvolatile metabolites and flavor components of cocoa liquor from different cacao genotypes (KW 516, SULAWESI 01, and ICCRI 09). According to a previous report(Dahiana Becerra et al., 2024), sensory notes such as floral, fruitiness, nuttiness, cocoa, browned roast, browned fruit, and wood are categorized as aroma components. Therefore, the second PLS model, shown in Fig. 5B, was constructed using volatile compounds (x, *n* = 66) as explanatory variables and sensory aroma attributes (y, n = 7), focusing on the aroma components of the cocoa liquor from different genotypes.

Based on the biplot (Fig. 5), ICCRI 09 was distinctly separated from Forastero (KW 516) owing to its markedly different sensory profile and metabolite composition. A 200-permutation test was conducted to validate the PLS model by comparing the Q^2^ values from the original dataset with the distribution of Q^2^ values generated by randomly assigning the original response variables (y-values) to individuals. The method described by Kim et al. (Kim et al., 2023) and Triba et al..(Triba et al., 2015) the criterion was that the Q^2^ and R^2^ values from the permuted datasets should be lower than those from the actual dataset. The permutation test demonstrated good predictability, as shown in Fig. S3, indicating that the sensory attributes were significantly correlated with the volatile and non-volatile metabolite profiles of the cocoa liquor.

As shown in Fig. 5A and supported by Table S3, which lists the top VIP metabolites, ICCRI 09 was characterized by outstanding sensory notes, such as floral, woody, cocoa, acidity, fresh fruit, browned roast, and global quality. These attributes are associated with nonvolatile compounds, including organic acids, sugars, and sugar derivatives. Floral and woody notes positively correlated with ribulose, trehalose, and glucono-1,5-lactone. Malic acid, citric acid, and 2-hydroxyglutaric acid are associated with acidic attributes, whereas fresh fruit is associated with psicose, citric acid, sorbitol, inositol, and malic acid. Cocoa notes were positively correlated with psicose, sorbitol, and ribulose content, whereas browned roasts were associated with inositol, galactinol, and sorbitol content. Global quality is associated with the galactinol, gentiobiose, and inositol levels. These findings confirm that ICCRI 09 has a rich flavor profile. A previous study reported that the combination of certain organic acids and sugars enhanced the balance between taste and flavor in cocoa(Holm et al., 1993). Additionally, this study found that SULAWESI 01, which is characterized by a nutty flavor, contains methionine, isoleucine, and glycine. A previous study has reported that glycine contributes to sweetness, whereas isoleucine and methionine contribute to bitterness(Bachmanov et al., 2016). This finding may explain the distinct flavor of SULAWESI 01 compared to that of ICCRI 09. However, no studies have yet reported an association between these compounds and the nutty flavor profile, although non-volatile compounds are known to influence the unique taste and aroma of cocoa(Castro-Alayo et al., 2019).

The Forastero variety KW 516, which exhibits basic sensory attributes, is dominated by bitterness and astringency. These attributes are associated with alkaloids and polyphenol compounds, such as theobromine, epicatechin, catechin, and stearic acid. This aligns with previous studies showing that theobromine, catechin, and epicatechin contribute to the bitterness and astringency of cocoa(Vázquez-Ovando et al., 2016). Despite the undesirable taste notes predominating in this variety, KW 516 also displayed a slightly browned fruit note, which correlated with sugar derivatives such as tartaric acid, mannitol, and erythritol. Although the chemical composition of non-volatile compounds provides valuable insights into flavor, it may not fully explain all the flavor components of cocoa liquor from different genotypes. Therefore, in this study, we explored the correlation between sensory attributes and volatile components.

A notable result from the correlation between the sensory profiles and volatile compounds, shown in Fig. 5B and Table S4, highlighted the top VIP metabolites that distinctly separated ICCRI 09 from KW 516 and SULAWESI 01. ICCRI 09 exhibited significantly higher levels of volatile compounds, including linalool, 1-phenylethyl alcohol, 2-propanol, and isobutyl acetate, which correlated with the flowery aroma. Additionally, this genotype showed significantly higher levels of benzaldehyde, 2,3-butanediol, 2-methyl-3-buten-2-ol, 3-hexanone, 2-pentanol, and isobutyl acetate, which correlated with fruity aroma. This study also found that 3-methylbutanal was associated with a cocoa/malty aroma. *Trans*-Linalool oxide, linked to woody aromas, and 2,3-butanedione and 2-ethyl-3,5-dimethylpyrazine, associated with browned roast notes, were also dominant in ICCRI 09. This finding aligns with a previous research that profiled the volatile components of cocoa and matched the aroma descriptors(Velásquez-Reyes et al., 2023)(Colonges, Jimenez, et al., 2022). However, no previous studies have quantitatively linked the relationship between these volatile compounds and sensory attributes across different cocoa genotypes. By establishing these quantitative correlations, this study highlights the distinctive sensory qualities of ICCRI 09 and its potential classification as high-quality cocoa.

Another remarkable finding was that SULAWESI 01 consistently grouped with nutty sensory profiles, which were linked to volatile compounds such as acetoin. However, a previous study found that acetoin is related to creamy aroma descriptors(Colonges, Jimenez, et al., 2022; Velásquez-Reyes et al., 2023). According to previous research, the nutty, almond-like aroma is influenced by phenylacetaldehyde and pyrazines such as 2-ethyl-3,5-dimethylpyrazine, 2,5-dimethylpyrazine, and 2,6-dimethylpyrazine. Because this study did not include a creamy aroma, future sensory analyses should incorporate buttery flavors to accurately confirm the correlation between this aroma and this genotype. Further results showed that KW 516, a Forastero genotype, was distinct from the Trinitario groups, which were dominated by bitterness and astringency. However, this genotype also exhibited a browned fruit aroma clustered with furfural, ethyl acetate, ethyl lactate, 2-heptanol, and 2-nonanone. This correlation aligns with previous studies stating that these volatile compounds are associated with fruity and browned fruit aromas(Velásquez-Reyes et al., 2023)(Colonges, Jimenez, et al., 2022). This study did not include undesired flavors, such as the sour vinegar aroma, in the sensory analysis; therefore, the correlation analysis did not highlight that acetic acid was the predominant aroma in this Forastero genotype.

Finally, the correlation between the sensory profiles and both volatile and non-volatile compounds of different cacao genotypes was confirmed using PLS regression analysis. Overall, the ICCRI 09 genotype shows potential as a candidate for high-quality cocoa production. This genotype has complex sensory attributes correlated with nonvolatile and volatile components, contributing to a balanced taste and distinct aroma. This study focuses on evaluating flavor components in only three cacao genotypes, which may limit the generalizability of the findings. Expanding the scope to include more genotypes and sensory attributes could provide deeper insights into cacao flavor diversity. Future research should also examine flavor changes throughout the post-harvest process to enhance the understanding and characterization of flavor development.

## 4 Conclusion

The relationship between the sensory attributes and metabolite profiles of cocoa liquor from different cacao genotypes was clarified through metabolomic analysis and sensory evaluation. The key compounds influencing flavor quality were identified through statistical correlations between sensory attributes and metabolomic data. Organic acids and sugars are associated with desirable flavor attributes, whereas polyphenols contribute to bitterness and astringency. Volatile compounds such as linalool, 1-phenylethyl alcohol, 2-propanol, and isobutyl acetate are associated with floral aromas, whereas benzaldehyde, 2,3-butanediol, 2-methyl-3-buten-2-ol, 3-hexanone, 2-pentanol, and isobutyl acetate are linked to fresh fruit aromas. Additionally, 3-methylbutanal was linked to a cocoa/malty aroma; trans-linalool oxide, associated with a woody aroma; and 2,3-butanedione and 2-Ethyl-3.5-dimethylpyrazine, correlated with browned roast, were significantly abundant in the ICCRI 09 genotype. These findings deepen our understanding of the complex flavors of cocoa from various genotypes by connecting sensory characteristics with chemical profiles. Furthermore, this integrative approach could be extended to other processed agricultural products to enhance flavor understanding and improvement.

## Ethical statement for sensory evaluation

This study involved the sensory evaluation of cocoa liquor, a commonly consumed food product. Prior to the evaluation, trained panelists at the Indonesian Coffee and Cocoa Research Institute (ICCRI) were informed of the study's purpose, procedures, and any potential risks. Informed consent was obtained from all participants to ensure transparency and voluntary participation. Ethical approval was not required by ICCRI for this type of sensory study involving professionally trained panelists.

## CRediT authorship contribution statement

**Enik Nurlaili Afifah:** Writing – review & editing, Writing – original draft, Visualization, Validation, Methodology, Investigation, Formal analysis, Data curation, Conceptualization. **Indah Anita Sari:** Writing – review & editing, Resources. **Agung Wahyu Susilo:** Resources. **Hendy Firmanto:** Writing – review & editing, Methodology. **Abdul Malik:** Methodology. **Eiichiro Fukusaki:** Writing – review & editing, Validation, Supervision, Resources, Data curation, Conceptualization. **Sastia Prama Putri:** Writing – review & editing, Validation, Supervision, Resources, Project administration, Methodology, Data curation, Conceptualization.

## Declaration of competing interest

The authors declare that they have no known competing financial interests or personal relationships that could have appeared to influence the work reported in this paper.

## Data Availability

The data supporting this study will be provided upon request.

## Appendix A Supplementary data

Supplementary material Image 1
