# Supplementary material for: Correlation between sensory attributes and Metabolomic profiles of cocoa liquor from different cacao genotypes
Source: Food Chem X. 2025 Apr 25;28:102498. doi: 10.1016/j.fochx.2025.102498 (PMC12136762; doi:10.1016/j.fochx.2025.102498)
Supplement: Supplementary file 1 — Supplementary material [file mmc1.docx]

**Correlation between Sensory Attributes and Metabolomic Profiles of Cocoa Liquor from Different Cacao Genotypes**

Short title: Correlation analysis of cocoa flavor

**Keyword:** Cocoa liquor; flavor; genotype; metabolomics; quality; sensory analysis; theobroma cacao.


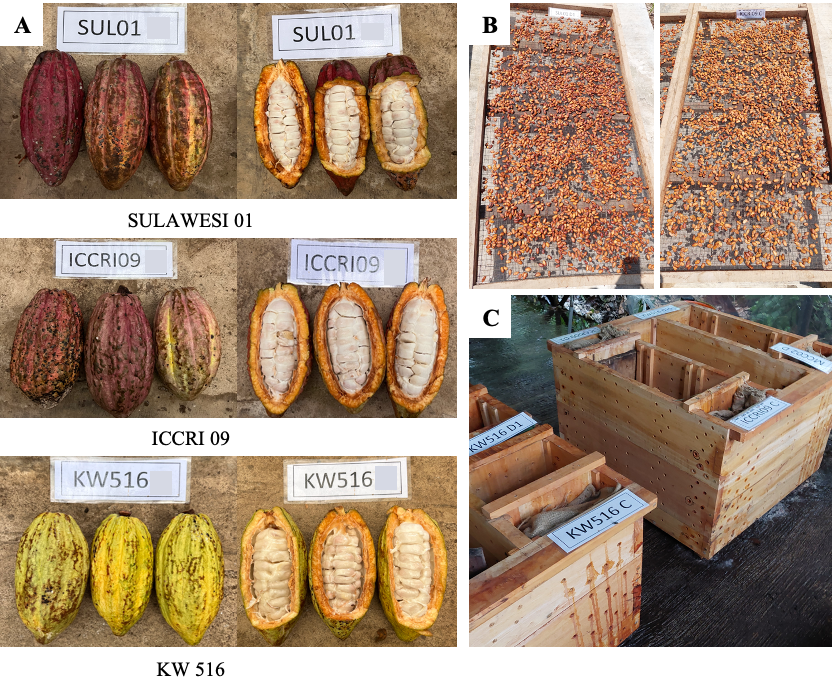


Figure S1. Cacao pod of each genotype (A), fermentation (B), and drying condition (C).

**(A)**

**(B)**

**(C)**


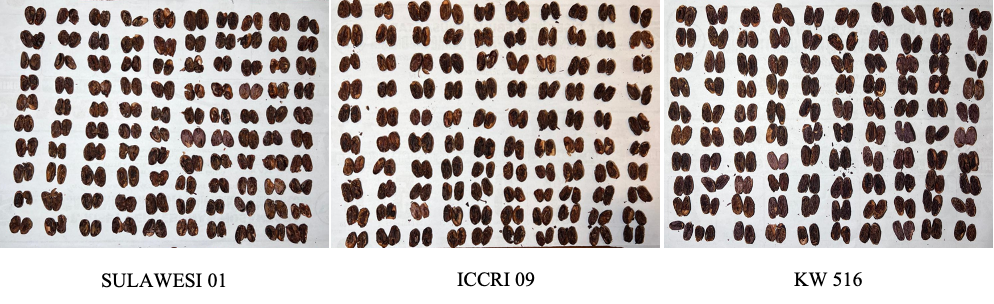


Figure S2. Bar chart of fermentation index (A), temperature during fermentation process (B), and cut test analysis (C).


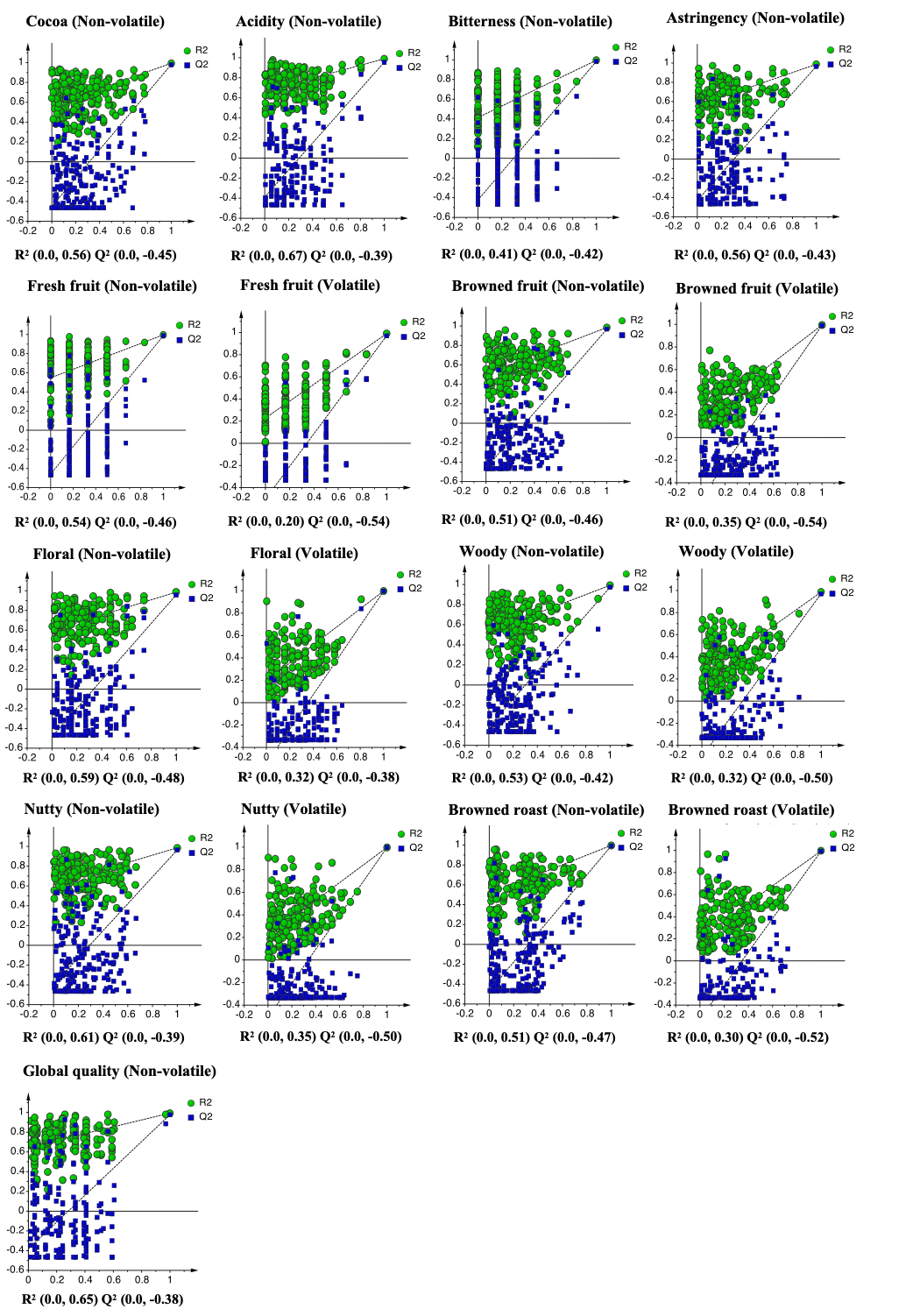


Figure S3. The result of validation by permutation test analysis (n = 200) of each sensory attribute

Table S1. Loading scores from PCA of non-volatile metabolites based on PC1 and PC2

| **No.** | **Metabolite name** | **PC1** | **Metabolite name** | **PC2** |
| --- | --- | --- | --- | --- |
| 1 | Citric acid | 0.168575 | Sucrose | 0.191519 |
| 2 | Phosphate | 0.168399 | Isoleucine | 0.191107 |
| 3 | Inositol | 0.168199 | Glycine | 0.18977 |
| 4 | Psicose | 0.168169 | Threonine | 0.189528 |
| 5 | 4-Aminobutyric acid | 0.166054 | Lactic acid | 0.186352 |
| 6 | Sorbitol | 0.165538 | Methionine | 0.174038 |
| 7 | 2-hydroxyglutaric acid | 0.163646 | Serine | 0.172321 |
| 8 | Malic acid | 0.161204 | Asparagine | 0.163095 |
| 9 | Ribulose | 0.15017 | Glucose | 0.162609 |
| 10 | Lyxose | 0.149419 | Proline | 0.157569 |
| 11 | Galactinol | 0.14528 | Caffeine | 0.154841 |
| 12 | 1,2,3-Butanetriol | 0.136614 | Shikimic acid | 0.152317 |
| 13 | 2-Dehydro gluconate | 0.134174 | Lysine | 0.147825 |
| 14 | Trehalose | 0.133211 | Glutamic acid | 0.147743 |
| 15 | Melibiose | 0.133001 | Aspartic acid | 0.145415 |
| 16 | Glucosamine | 0.132336 | Adenine | 0.139841 |
| 17 | Fructose | 0.131605 | Fumaric acid | 0.136513 |
| 18 | Threitol | 0.127764 | Xylitol | 0.135274 |
| 19 | Glyceric acid | 0.126631 | Gentiobiose | 0.13504 |
| 20 | Glycolic acid | 0.124458 | Glucosamine | 0.132605 |
| 21 | Gentiobiose | 0.121511 | Fructose | 0.128594 |
| 22 | Glutamic acid | 0.115479 | UDP-N-acetylglucosamine | 0.12851 |
| 23 | Mannose | 0.112784 | Mannose | 0.126227 |
| 24 | Glucono-1,5-lactone | 0.112596 | Tyrosine | 0.118673 |
| 25 | Succinic acid | 0.109885 | 2-Dehydro gluconate | 0.107226 |
| 26 | Glycerol | 0.108968 | Galactinol | 0.106898 |
| 27 | Glucose | 0.106133 | Beta-Alanine | 0.103239 |
| 28 | Saccharic acid | 0.105803 | Palmitic acid | 0.075388 |
| 29 | Arabitol | 0.104048 | Stearic acid | 0.070386 |
| 30 | Aspartic acid | 0.103154 | Alanine | 0.068851 |
| 31 | Beta-Alanine | 0.081336 | 4-Aminobutyric acid | 0.052619 |
| 32 | Asparagine | 0.077276 | Epicatechin | 0.033851 |
| 33 | Proline | 0.073822 | Inositol | 0.026943 |
| 34 | Threonic acid | 0.0649 | Malic acid | 0.006623 |
| 35 | Sucrose | 0.051151 | 2,3 Buatanediol | -0.00181 |
| 36 | Serine | 0.04474 | Phenylalanine | -0.00404 |
| 37 | Alanine | 0.039811 | Phosphate | -0.02041 |
| 38 | Adenine | 0.025605 | Sorbitol | -0.02082 |
| 39 | Lactic acid | 0.013894 | Citric acid | -0.03231 |
| 40 | Glycine | 0.011432 | Valine | -0.04034 |
| 41 | UDP-N-acetylglucosamine | -0.00205 | Psicose | -0.04145 |
| 42 | Pyroglutamic acid | -0.00324 | Meso erythritol | -0.04467 |
| 43 | Threonine | -0.00351 | 2-Aminoethanol | -0.05079 |
| 44 | Valine | -0.01275 | Myo-Inositol | -0.05267 |
| 45 | Shikimic acid | -0.0262 | 3-Phenyllactic acid | -0.05267 |
| 46 | Tartaric acid | -0.03505 | 2-hydroxyglutaric acid | -0.05493 |
| 47 | Isoleucine | -0.04864 | Xylonic acid | -0.05891 |
| 48 | Xylonic acid | -0.07489 | Threitol | -0.06434 |
| 49 | Leucine | -0.08121 | Leucine | -0.06722 |
| 50 | Caffeine | -0.08128 | Catechine | -0.0677 |
| 51 | Methionine | -0.08358 | Theobromine | -0.07506 |
| 52 | Fumaric acid | -0.1043 | Erythritol | -0.08399 |
| 53 | 2,3 Buatanediol | -0.1055 | Mannitol | -0.09451 |
| 54 | Palmitic acid | -0.10591 | Threonic acid | -0.09569 |
| 55 | L-Alanine | -0.10704 | Saccharic acid | -0.09624 |
| 56 | Stearic acid | -0.11118 | Ribulose | -0.09716 |
| 57 | Xylitol | -0.1191 | Lyxose | -0.1018 |
| 58 | Lysine | -0.12174 | Arabinopyranose | -0.10691 |
| 59 | Theobromine | -0.12769 | Glyceric acid | -0.10738 |
| 60 | Tyrosine | -0.13873 | Melibiose | -0.11737 |
| 61 | Arabinopyranose | -0.14546 | Trehalose | -0.1182 |
| 62 | Phenylalanine | -0.15178 | Glycerol | -0.1198 |
| 63 | Mannitol | -0.1521 | 1,2,3-Butanetriol | -0.1208 |
| 64 | Erythritol | -0.15267 | Glycolic acid | -0.14113 |
| 65 | Catechine | -0.16137 | Succinic acid | -0.14612 |
| 66 | Myo-Inositol | -0.16474 | Glucono-1,5-lactone | -0.15701 |
| 67 | Meso erythritol | -0.1655 | Arabitol | -0.15874 |
| 68 | 3-Phenyllactic acid | -0.16665 | L-Alanine | -0.16302 |
| 69 | 2-Aminoethanol | -0.16678 | Pyroglutamic acid | -0.16859 |
| 70 | Epicatechin | -0.16935 | Tartaric acid | -0.20133 |

Table S2. Loading scores from PCA of volatile compounds based on PC1 and PC2

| **No.** | **Metabolite name** | **PC1** | **Metabolite name** | **PC2** |
| --- | --- | --- | --- | --- |
| 1 | Benzaldehyde | 0.159444 | 2,6-Dimethylpyrazine | 0.216446 |
| 2 | Butyric acid | 0.159356 | 2,5-dimethylpyrazine | 0.215002 |
| 3 | 2,3-Butanediol | 0.158885 | 3-Methoxybutyl Acetate | 0.213148 |
| 4 | Trans-Linalool oxide | 0.158371 | Gamma-Butyrolactone | 0.212129 |
| 5 | Pentanoic acid | 0.155409 | Ethyl Acetate | 0.212124 |
| 6 | 2-methyl-3-buten-2-ol | 0.154958 | 2-Methylpyrazine | 0.203628 |
| 7 | Butanoic acid | 0.154058 | Furfural | 0.201488 |
| 8 | 1-Pentanol | 0.148373 | 3-Methyl-1-butanol | 0.199941 |
| 9 | Phenylacetaldehyde | 0.147243 | 2-Methyl-1-butanol | 0.193756 |
| 10 | 3-Hexanone | 0.147209 | Ethanol | 0.190166 |
| 11 | Trans-2-Nonenal | 0.146877 | Ethyl Lactate | 0.18 |
| 12 | Butyraldehyde | 0.146782 | 3-methylbutyl acetate | 0.174676 |
| 13 | 2,4,7-Octanetrione | 0.146457 | Ethyl Hexanoate | 0.174413 |
| 14 | 2,2,6-Trimethyloctane | 0.145705 | 2-Methylbutyraldehyde | 0.138915 |
| 15 | Ethyl benzene | 0.145333 | Linalool | 0.138839 |
| 16 | isobutyl acetate | 0.145296 | 2-Ethyl-3.5-dimethylpyrazine | 0.13812 |
| 17 | Isoamyl Ether | 0.143921 | 3-Carene | 0.129572 |
| 18 | 3-methylbutanal | 0.143157 | Acetol | 0.128173 |
| 19 | Valeraldehyde | 0.142897 | 2-Pentanol | 0.126328 |
| 20 | 1-Phenylethyl alcohol | 0.137259 | 2-propanol | 0.118388 |
| 21 | 2-Prphyl1-Pentanol | 0.135693 | cis-B-Ocimene | 0.108232 |
| 22 | 2-propanol | 0.131202 | 2,3,5-Trimethylpyrazine | 0.106983 |
| 23 | 2-Phenylpropionaldehyde | 0.130938 | 1-Butanol | 0.105547 |
| 24 | 2-Pentanol | 0.130307 | 2,3,5,6-Tetramethylpyrazine | 0.103948 |
| 25 | Acetol | 0.129135 | 3-methylbutanal | 0.095987 |
| 26 | 2,3-butanedione | 0.125188 | 1-Phenylethyl alcohol | 0.090383 |
| 27 | 2-Methylbutyraldehyde | 0.121775 | 2-Methyl-1-propanol | 0.089001 |
| 28 | Linalool | 0.118093 | 2-Butanone | 0.088397 |
| 29 | 2-heptanone | 0.116118 | Isoamyl Ether | 0.088128 |
| 30 | 2-Methyl-1-propanol | 0.109547 | isobutyl acetate | 0.087767 |
| 31 | Nonanal | 0.108794 | 3-Acetoxy-2-butanone | 0.084766 |
| 32 | 4-Methyl-valeric acid | 0.106639 | 2,2,6-Trimethyloctane | 0.0833 |
| 33 | 1-Butanol | 0.095333 | 2-Heptanol | 0.078883 |
| 34 | 3-methylbutyl acetate | 0.085876 | 2-Nonanone | 0.076891 |
| 35 | Isobutyric Acid | 0.081141 | 3-Hexanone | 0.075108 |
| 36 | 2-Methyl-1-butanol | 0.06728 | 2-Prphyl1-Pentanol | 0.068765 |
| 37 | 3-Methyl-1-butanol | 0.059235 | Butanoic acid | 0.060226 |
| 38 | 2-Methylpyrazine | 0.047502 | Phenylacetaldehyde | 0.056679 |
| 39 | Ethyl Hexanoate | 0.04358 | Pentanoic acid | 0.052379 |
| 40 | Gamma-Butyrolactone | 0.03367 | 2-methyl-3-buten-2-ol | 0.052363 |
| 41 | 2,5-dimethylpyrazine | 0.005902 | Methanol | 0.050354 |
| 42 | 2,6-Dimethylpyrazine | -0.00551 | 2-Acetylfuran | 0.049934 |
| 43 | 3-Methoxybutyl Acetate | -0.00719 | Butyraldehyde | 0.0435 |
| 44 | 1-Propanol | -0.01155 | Ethyl Octanoate | 0.039734 |
| 45 | Ethyl Acetate | -0.01584 | Pivalic Acid | 0.038951 |
| 46 | Ethanol | -0.02282 | Acetic acid | 0.032253 |
| 47 | Ethyl Octanoate | -0.05413 | 2,3-Butanediol | 0.026993 |
| 48 | Furfural | -0.05481 | Benzaldehyde | 0.014803 |
| 49 | 2-Acetylfuran | -0.07272 | Trans-Linalool oxide | 0.013811 |
| 50 | Ethyl Lactate | -0.07543 | Butyric acid | -0.01342 |
| 51 | Acetoin | -0.08921 | Isoamyl Lactate | -0.01935 |
| 52 | 2-Ethyl-3.5-dimethylpyrazine | -0.12346 | Benzonitrile | -0.02831 |
| 53 | Benzonitrile | -0.12612 | Propanoic acid | -0.02857 |
| 54 | 3-Carene | -0.1278 | 1-Pentanol | -0.06692 |
| 55 | cis-B-Ocimene | -0.13938 | Trans-2-Nonenal | -0.07033 |
| 56 | 2,3,5-Trimethylpyrazine | -0.13994 | Ethyl benzene | -0.0775 |
| 57 | 2,3,5,6-Tetramethylpyrazine | -0.14068 | Valeraldehyde | -0.07765 |
| 58 | 2-Butanone | -0.14225 | 2,4,7-Octanetrione | -0.08668 |
| 59 | 3-Acetoxy-2-butanone | -0.14621 | 2-heptanone | -0.09898 |
| 60 | Methanol | -0.14687 | 4-Methyl-valeric acid | -0.11414 |
| 61 | 2-Heptanol | -0.14995 | 2,3-butanedione | -0.1156 |
| 62 | 2-Nonanone | -0.15016 | 2-Phenylpropionaldehyde | -0.12359 |
| 63 | Isoamyl Lactate | -0.15259 | Nonanal | -0.14812 |
| 64 | Acetic acid | -0.15357 | Acetoin | -0.16097 |
| 65 | Pivalic Acid | -0.15739 | Isobutyric Acid | -0.17823 |
| 66 | Propanoic acid | -0.15852 | 1-Propanol | -0.21098 |

Table S3. List of the top VIP non-volatile metabolites highlighted in the correlation biplot (Figure 5)

| **Response variable: Cocoa** | | | |
| --- | --- | --- | --- |
| **No.** | **Metabolite name** | **VIP score** | **Coefficient** |
| 1 | Psicose | 1.442 | 0.135 |
| 2 | Sorbitol | 1.389 | 0.287 |
| 3 | Ribulose | 1.357 | 0.312 |
| 4 | Inositol | 1.349 | 0.235 |
| 5 | Lyxose | 1.340 | 0.382 |
| **Response variable: Acidity** | | | |
| **No.** | **Metabolite name** | **VIP score** | **Coefficient** |
| 1 | Malic acid | 1.325 | 0.366 |
| 2 | Citric acid | 1.200 | 0.191 |
| 3 | 2-hydroxyglutaric acid | 1.124 | 0.204 |
| 4 | Glutamic acid | 1.300 | 0.207 |
| 5 | Aspartic acid | 1.138 | 0.444 |
| **Response variable: Astringency** | | | |
| **No.** | **Metabolite name** | **VIP score** | **Coefficient** |
| 1 | Tartaric acid | 1.709 | 0.345 |
| 2 | Pyroglutamic acid | 1.526 | 0.524 |
| 3 | Lactic acid | 1.487 | 0.735 |
| 4 | Tartaric acid | 1.709 | 0.345 |
| **Response variable: Bitterness** | | | |
| **No.** | **Metabolite name** | **VIP score** | **Coefficient** |
| 1 | Catechine | 1.330 | 0.308 |
| 2 | Glycine | 1.285 | 0.814 |
| 3 | Theobromine | 1.029 | 0.394 |
| 4 | Epicatechin | 1.028 | 0.654 |
| **Response variable: Fresh fruit** | | | |
| **No.** | **Metabolite name** | **VIP score** | **Coefficient** |
| 1 | Psicose | 1.431 | 0.153 |
| 2 | Citric acid | 1.429 | 0.174 |
| 3 | Sorbitol | 1.386 | 0.282 |
| 4 | Inositol | 1.372 | 0.215 |
| 5 | Malic acid | 1.359 | 0.245 |
| **Response variable: Browned fruit** | | | |
| **No.** | **Metabolite name** | **VIP score** | **Coefficient** |
| 1 | Tartaric acid | 1.616 | 0.452 |
| 2 | Mannitol | 1.291 | 0.458 |
| 3 | Erythritol | 1.251 | 0.502 |
| 4 | Isoleucine | 1.177 | 0.887 |
| 5 | Caffeine | 1.118 | 1.026 |
| **Response variable: Floral** | | | |
| **No.** | **Metabolite name** | **VIP score** | **Coefficient** |
| 1 | Ribulose | 1.497 | 0.237 |
| 2 | Trehalose | 1.479 | 0.512 |
| 3 | Glycolic acid | 1.477 | 0.450 |
| 4 | Glucono-1,5-lactone | 1.460 | 0.410 |
| 5 | Lyxose | 1.452 | 0.296 |
| **Response variable: Woody** | | | |
| **No.** | **Metabolite name** | **VIP score** | **Coefficient** |
| 1 | Ribulose | 1.451 | 0.286 |
| 2 | Psicose | 1.441 | 0.110 |
| 3 | Lyxose | 1.421 | 0.356 |
| 4 | Trehalose | 1.374 | 0.493 |
| 5 | Glycolic acid | 1.347 | 0.484 |
| **Response variable: Nutty** | | | |
| **No.** | **Metabolite name** | **VIP score** | **Coefficient** |
| 1 | Methionine | 1.501 | 0.360 |
| 2 | Isoleucine | 1.410 | 0.850 |
| 3 | Glycine | 1.278 | 0.784 |
| 4 | Sucrose | 1.262 | 0.487 |
| 5 | Pyroglutamic acid | 1.239 | 0.764 |
| **Response variable: Browned Roast** | | | |
| **No.** | **Metabolite name** | **VIP score** | **Coefficient** |
| 1 | Inositol | 1.416 | 0.191 |
| 2 | Galactinol | 1.389 | 0.219 |
| 3 | Sorbitol | 1.305 | 0.266 |
| 4 | Glucose | 1.175 | 0.298 |
| 5 | Glutamic acid | 1.174 | 0.267 |
| **Response variable: Global Quality** | | | |
| **No.** | **Metabolite name** | **VIP score** | **Coefficient** |
| 1 | Galactinol | 1.468 | 0.191 |
| 2 | Gentiobiose | 1.400 | 0.189 |
| 3 | Inositol | 1.383 | 0.204 |
| 4 | Glucose | 1.337 | 0.224 |
| 5 | Malic acid | 1.325 | 0.366 |

Table S4. List of the top VIP volatile metabolites highlighted in the correlation biplot (Figure 5)

| **Response variable: Fresh fruit** | | | |
| --- | --- | --- | --- |
| **No.** | **Metabolite name** | **VIP score** | **Coefficient** |
| 1 | 2,3-Butanediol | 1.311 | 0.101 |
| 2 | Benzaldehyde | 1.304 | 0.118 |
| 3 | 2-methyl-3-buten-2-ol | 1.290 | 0.181 |
| 4 | 3-methylbutanal | 1.249 | 0.279 |
| 5 | isobutyl acetate | 1.230 | 0.277 |
| 6 | 3-Hexanone | 1.226 | 0.316 |
| 7 | 2-Pentanol | 1.166 | 0.353 |
| **Response variable: Browned fruit** | | | |
| **No.** | **Metabolite name** | **VIP score** | **Coefficient** |
| 1 | Furfural | 1.670 | 0.274 |
| 2 | Ethyl Lactate | 1.601 | 0.277 |
| 3 | Ethyl Acetate | 1.594 | 0.475 |
| 4 | 2-Heptanol | 1.131 | 0.506 |
| 5 | 2-Nonanone | 1.116 | 0.521 |
| **Response variable: Floral** | | | |
| **No.** | **Metabolite name** | **VIP score** | **Coefficient** |
| 1 | Linalool | 1.406 | 0.420 |
| 2 | 2-propanol | 1.370 | 0.407 |
| 3 | isobutyl acetate | 1.360 | 0.243 |
| 4 | 1-Phenylethyl alcohol | 1.287 | 0.407 |
| **Response variable: Woody** | | | |
| **No.** | **Metabolite name** | **VIP score** | **Coefficient** |
| 1 | Ribulose | 1.450 | 0.286 |
| 2 | Trehalose | 1.374 | 0.493 |
| 3 | Glucono-1,5-lactone | 1.296 | 0.480 |
| **Response variable: Nutty** | | | |
| **No.** | **Metabolite name** | **VIP score** | **Coefficient** |
| 1 | Acetoin | 1.554 | 0.366 |
| 2 | 2,5-dimethylpyrazine | 1.549 | 0.409 |
|  | 2-Methylbutyraldehyde | 1.491 | 0.204 |
| **Response variable: Browned Roast** | | | |
| **No.** | **Metabolite name** | **VIP score** | **Coefficient** |
| 1 | 2-Ethyl-3.5-dimethylpyrazine | 1.174 | 0.232 |
| 2 | 2,3-butanedione | 1.160 | 0.249 |
| **Response variable: Cocoa** | | | |
| **No.** | **Metabolite name** | **VIP score** | **Coefficient** |
| 1 | 3-Methylbutanal | 1.283 | 0.269 |
| 2 | Phenylacetaldehyde | 1.250 | 0.188 |

Table S5. All volatile metabolites detected in this study and the aroma descriptor

| **Metabolites** | **Group** | **ICCRI09** | **SUL01** | **KW516** | **Aroma description** |
| --- | --- | --- | --- | --- | --- |
| 4-Methyl-valeric acid | Acid | 27474.22 | 30701.69 | 20176.28 |  |
| Acetic acid | Acid | 2836865.00 | 3066434.33 | 3896622.33 | Sour, vinegar |
| Butyric acid | Acid | 891739.40 | 773195.07 | 514922.60 |  |
| Isobutyric Acid | Acid | 750288.97 | 926363.63 | 638143.13 |  |
| Pivalic Acid | Acid | 26492.54 | 45377.54 | 109705.13 |  |
| Propanoic acid | Acid | 141895.70 | 4341779.33 | 8458767.00 | Acidic, pungent, cheese |
| Pentanoic acid | Acid | 174487.27 | 113795.07 | 72065.59 |  |
| Butanoic acid | Acid | 455453.77 | 278576.10 | 174276.90 | Acetic, cheese, butter |
| 1-Butanol | Alcohol | 2109.79 | 835.11 | 853.20 |  |
| 1-Pentanol | Alcohol | 6512.45 | 6278.89 | 3217.36 | Fruity, green |
| 2-Heptanol | Alcohol | 2465.40 | 2864.66 | 20801.27 | Floral, earthy, fruity, citrus |
| 2-Methyl-1-propanol | Alcohol | 3836.12 | 3080.08 | 2985.43 | Wine |
| 2-Methyl-3-buten-2-ol | Alcohol | 7695.92 | 4928.49 | 3015.27 | Herbal, earth, oily |
| 2-Pentanol | Alcohol | 28600.52 | 7434.69 | 6596.78 | Green, fruity, sweet, fusel oil |
| 2-propanol | Alcohol | 13371.46 | 4319.32 | 3562.14 |  |
| 2-Prphyl-1-Pentanol | Alcohol | 2430.73 | 1888.68 | 1649.83 |  |
| 3-Methyl-1-butanol | Alcohol | 9155.58 | 3171.07 | 6206.91 | Fruity |
| 2-Methyl-1-butanol | Alcohol | 3332.54 | 1306.41 | 2234.99 | Fruity |
| Ethanol | Alcohol | 13605.12 | 10336.21 | 13835.87 | Undesirable note |
| Methanol | Alcohol | 3795.04 | 4318.01 | 7490.61 |  |
| 1-Phenylethyl alcohol | Alcohol | 9136.40 | 6805.04 | 6147.89 | Honey, flowery |
| 2,3-Butanediol | Alcohol | 11073.48 | 8559.69 | 5934.05 | Fruity, creamy |
| 1-Propanol | Alcohol | 12076.86 | 33940.21 | 16917.02 | Pungent, sweet, candy |
| 2-Butanone | Aldehyde | 6002.41 | 5859.16 | 10514.38 |  |
| 2-Methylbutyraldehyde | Aldehyde | 40968.83 | 20463.19 | 21502.55 |  |
| 2-Phenylpropionaldehyde | Aldehyde | 12596.72 | 15768.97 | 2226.83 |  |
| 3-methylbutanal | Aldehyde | 99225.65 | 55473.40 | 44285.47 | Malty, chocolate |
| Benzaldehyde | Aldehyde | 49795.71 | 38869.34 | 24813.59 | Fruity, almond |
| Butyraldehyde | Aldehyde | 48286.21 | 30808.61 | 17499.33 |  |
| Nonanal | Aldehyde | 10360.02 | 12866.85 | 6574.69 | Citrus |
| Phenylacetaldehyde | Aldehyde | 52218.04 | 39713.27 | 32272.91 | Honey, nutty |
| Trans-2-Nonenal | Aldehyde | 7168.45 | 7078.00 | 3850.42 |  |
| Valeraldehyde | Aldehyde | 8481.40 | 8473.82 | 4535.76 |  |
| 3-Hexanone | Aldehyde | 31031.93 | 17180.09 | 11204.75 | Wine, fruity |
| 3-Methoxybutyl Acetate | Ester | 4793.40 | 2397.68 | 4585.74 |  |
| 3-methylbutyl acetate | Ester | 3342.83 | 1818.25 | 2322.23 | Fruity |
| Ethyl Acetate | Ester | 15917.04 | 6565.02 | 15482.69 | Fruity, sweet |
| Ethyl Lactate | Ester | 5587.65 | 3855.73 | 6597.11 | Fruity |
| Ethyl Octanoate | Ester | 2574.55 | 2539.28 | 2849.37 | Fruity, apricot |
| Isoamyl Ether | Ester | 7280.34 | 3929.83 | 2868.78 |  |
| Isoamyl Lactate | Ester | 2777.68 | 7662.88 | 13103.81 |  |
| isobutyl acetate | Ester | 9113.04 | 5215.37 | 3925.92 | Fruity, floral |
| Ethyl Hexanoate | Ester | 1264.17 | 914.35 | 1112.10 | Fruity, green, floral |
| Ethyl acetate | Ester | 185075.73 | 615666.20 | 1474126.33 | Fruity, sweet |
| 2-Acetylfuran | Furans | 206.05 | 120.73 | 1397.09 |  |
| Furfural | Furans | 34553.79 | 10444.74 | 41343.97 | Fruity, flowery, roasted, almond |
| 2-heptanone | Ketone | 9020.01 | 9695.58 | 6084.89 | Fruity |
| 2-Nonanone | Ketone | 2796.13 | 3742.86 | 30285.89 | Fruity, sweet |
| 2,3-butanedione | Ketone | 44177.18 | 47108.68 | 32938.49 | Buttery, creamy |
| 3-Acetoxy-2-butanone | Ketone | 17121.53 | 17113.53 | 29369.83 |  |
| Gamma-Butyrolactone | Lactone | 45358.14 | 13835.52 | 34595.18 | Caramel, sweet, creamy |
| Linalool | Lactones | 35404.76 | 13832.96 | 15490.94 | Rose, floral, green |
| Benzonitrile | Nitriles | 484.94 | 571.60 | 655.00 |  |
| 3-Carene | Others | 1513.08 | 655.24 | 3873.36 |  |
| Acetoin | Others | 483727.53 | 632059.07 | 590896.07 | Buttery, creamy |
| Acetol | Others | 48631.63 | 26657.56 | 26014.91 |  |
| cis-B-Ocimene | Others | 1703.02 | 883.12 | 6717.77 | Floral, herbal |
| Ethyl benzene | Others | 9008.91 | 8864.91 | 4533.66 | Fruity |
| Trans-Linalool oxide | Others | 9370.89 | 6470.68 | 2854.29 | Floral, honey, woody, lemon peel |
| 2,4,7-Octanetrione | Others | 23198.68 | 23374.28 | 10463.43 |  |
| 2-Ethyl-3.5-dimethylpyrazine | Pyrazine | 11553.88 | 5301.21 | 25750.18 | Caramel, roasted, nutty |
| 2-Methylpyrazine | Pyrazine | 6843.38 | 2542.18 | 5000.96 | Nutty, caramel, roasted |
| 2,3,5-Trimethylpyrazine | Pyrazine | 53601.82 | 34586.69 | 178249.40 | Nutty, cocoa |
| 2,3,5,6-Tetramethylpyrazine | Pyrazine | 147438.80 | 99182.86 | 504097.50 | Nutty, cocoa |
| 2,5-dimethylpyrazine | Pyrazine | 7476.07 | 3166.78 | 6682.65 | Caramel, roasted, nutty |
| 2,6-Dimethylpyrazine | Pyrazine | 12923.46 | 5886.77 | 12238.68 | Caramel, roasted, nutty |

Note: The flavor descriptor was based on the previous studies (Yang et al., 2024; Velásquez-Reyes et al., 2023; Akoa et al., 2023; Colonges et al., 2022; Bastos et al., 2019)

List of Annotated Metabolites Obtained from GC/MS analysis

| No. | Metabolite name | RT^a^ (min) | RI^b^ | Quant mass (m/z) | Similarity (%) | Library |
| --- | --- | --- | --- | --- | --- | --- |
| 1 | 2,3 Buatanediol | 4.189 | 1047.43 | 117.0782 | 92 | NIST 20s |
| 2 | Lactic acid | 4.438 | 1066.08 | 147.0718 | 99.9 | In-house |
| 3 | Glycolic acid | 4.629 | 1080.47 | 147.0697 | 96.4 | In-house |
| 4 | L-Alanine | 5.02 | 1109.24 | 147.05 | 90 | NIST 20s |
| 5 | Alanine | 5.025 | 1109.56 | 116.0974 | 96.4 | In-house |
| 6 | Isoleucine | 6.027 | 1180.29 | 86.0423 | 83.6 | In-house |
| 7 | Valine | 6.661 | 1225.34 | 144.1231 | 98 | In-house |
| 8 | Serine | 7.217 | 1265.06 | 116.0766 | 93.3 | In-house |
| 9 | 2-Aminoethanol | 7.376 | 1276.4 | 174.0949 | 98 | In-house |
| 10 | Leucine | 7.458 | 1282.24 | 158.109 | 93.4 | In-house |
| 11 | Phosphate | 7.498 | 1285.13 | 299.0936 | 99.1 | In-house |
| 12 | Glycerol | 7.538 | 1287.95 | 147.0782 | 99.2 | In-house |
| 13 | Threonine | 7.749 | 1303.15 | 117.0919 | 86.7 | In-house |
| 14 | Proline | 7.778 | 1305.32 | 142.1192 | 93.7 | In-house |
| 15 | 1,2,3-Butanetriol | 7.807 | 1307.55 | 117.0727 | 90 | NIST 20s |
| 16 | Glycine | 7.928 | 1316.6 | 174.0987 | 99.3 | In-house |
| 17 | Succinic acid | 7.973 | 1319.9 | 147.0697 | 96.7 | In-house |
| 18 | Glyceric acid | 8.315 | 1345.51 | 147.075 | 94.2 | In-house |
| 19 | Beta-Alanine | 9.58 | 1442.16 | 174.0965 | 81.2 | In-house |
| 20 | Malic acid | 10.368 | 1504.4 | 147.0705 | 97.8 | In-house |
| 21 | Threitol | 10.61 | 1524.53 | 147.0667 | 90.7 | In-house |
| 22 | Methionine | 10.679 | 1530.26 | 176.0689 | 84.5 | In-house |
| 23 | Pyroglutamic acid | 10.701 | 1532.09 | 156.0766 | 95.4 | In-house |
| 24 | Meso erythritol | 10.706 | 1532.5 | 147.0714 | 97.1 | In-house |
| 25 | Aspartic acid | 10.753 | 1536.43 | 232.0872 | 98 | In-house |
| 26 | 4-Aminobutyric acid | 10.821 | 1542.06 | 174.0962 | 99.5 | In-house |
| 27 | Threonic acid | 11.351 | 1586.01 | 147.072 | 88.6 | In-house |
| 28 | 2-hydroxyglutaric acid | 11.402 | 1590.27 | 129.096 | 83.5 | In-house |
| 29 | 3-Phenyllactic acid | 11.479 | 1596.69 | 193.0736 | 93 | In-house |
| 30 | Glutamic acid | 11.921 | 1635.25 | 246.1103 | 98.2 | In-house |
| 31 | Phenylalanine | 11.968 | 1639.34 | 218.0667 | 99 | In-house |
| 32 | Tartaric acid | 12.265 | 1665.33 | 147.0676 | 97.9 | In-house |
| 33 | Asparagine | 12.514 | 1687.13 | 116.0875 | 95.9 | In-house |
| 34 | Lyxose | 12.591 | 1693.86 | 103.0125 | 87.1 | In-house |
| 35 | Ribulose | 12.761 | 1709.25 | 147.0673 | 92.1 | In-house |
| 36 | Xylitol | 13.113 | 1741.68 | 217.066 | 87.1 | In-house |
| 37 | Arabitol | 13.248 | 1754.12 | 217.0818 | 99.1 | In-house |
| 38 | UDP-N-acetylglucosamine | 13.698 | 1795.53 | 129.1036 | 80.5 | In-house |
| 39 | Xylonic acid | 13.753 | 1800.61 | 147.0659 | 89.9 | In-house |
| 40 | Shikimic acid | 14.084 | 1832.64 | 204.0603 | 81.8 | In-house |
| 41 | Citric acid | 14.228 | 1846.59 | 273.1042 | 97.8 | In-house |
| 42 | Caffeine | 14.272 | 1850.8 | 194.0395 | 93.4 | In-house |
| 43 | Theobromine | 14.454 | 1868.4 | 180.041 | 98 | NIST 20s |
| 44 | Adenine | 14.559 | 1878.53 | 264.1051 | 87.5 | In-house |
| 45 | Psicose | 14.737 | 1895.76 | 103.0344 | 87.3 | In-house |
| 46 | Fructose | 14.898 | 1911.77 | 103.0375 | 99.6 | In-house |
| 47 | Mannose | 15.041 | 1926.21 | 160.0739 | 90.2 | In-house |
| 48 | 2-Dehydro gluconate | 15.101 | 1932.37 | 103.025 | 96.1 | In-house |
| 49 | Glucose | 15.16 | 1938.31 | 205.0759 | 99.1 | In-house |
| 50 | Lysine | 15.196 | 1941.99 | 174.0968 | 96.7 | In-house |
| 51 | Glucosamine | 15.296 | 1952.03 | 203.0682 | 87.7 | In-house |
| 52 | Tyrosine | 15.358 | 1958.34 | 218.0692 | 95.7 | In-house |
| 53 | Fumaric acid | 15.386 | 1961.11 | 144.1141 | 95 | NIST 20s |
| 54 | Mannitol | 15.49 | 1971.67 | 205.0757 | 99.3 | In-house |
| 55 | Sorbitol | 15.565 | 1979.23 | 205.0705 | 93.3 | In-house |
| 56 | Erythritol | 15.879 | 2011.48 | 205.0841 | 89 | NIST 20s |
| 57 | Glucono-1,5-lactone | 16.199 | 2045.43 | 147.0734 | 98.5 | In-house |
| 58 | Palmitic acid | 16.231 | 2048.75 | 313.275 | 94.3 | In-house |
| 59 | Saccharic acid | 16.337 | 2060.02 | 147.0774 | 86.7 | In-house |
| 60 | Myo-Inositol | 16.429 | 2069.74 | 217.0797 | 93 | NIST 20s |
| 61 | Inositol | 17.002 | 2131.71 | 217.0782 | 99.1 | In-house |
| 62 | Stearic acid | 18.025 | 2246.54 | 117.0349 | 90.1 | In-house |
| 63 | Sucrose | 21.729 | 2710.71 | 361.1819 | 98.2 | In-house |
| 64 | Trehalose | 22.493 | 2817.11 | 361.1758 | 96.2 | In-house |
| 65 | Epicatechin | 23.071 | 2900.11 | 368.1757 | 98.2 | In-house |
| 66 | Catechine | 23.234 | 2924.12 | 368.1676 | 85 | NIST 20s |
| 67 | Gentiobiose | 23.27 | 2929.31 | 204.05 | 87.2 | In-house |
| 68 | Melibiose | 23.48 | 2960.21 | 204.066 | 74.4 | In-house |
| 69 | Galactinol | 24.22 | 3072.19 | 204.0576 | 82.6 | In-house |
| 70 | Arabinopyranose | 24.936 | 3184.11 | 204.0621 | 95 | NIST 20s |

Remarks^: a^ (RT) Retention time in minutes

^b^ (RI) Retention Indices, calculated by standard alkane mixture (C10-C40).

List of Annotated Metabolites Obtained from HS-SPME Arrow GC/MS analysis

| No. | Metabolite name | RT^a^ (min) | RI^b^ | Quant mass (m/z) | Similarity (%) | Library |
| --- | --- | --- | --- | --- | --- | --- |
| 1 | Butyraldehyde | 4.623 | 176.31 | 43.08 | 84.5 | In-house |
| 2 | Ethyl Acetate | 6.087 | 220.56 | 43.04445 | 83.6 | In-house |
| 3 | Methanol | 6.323 | 229.41 | 31.05 | 80.5 | In-house |
| 4 | 2-Butanone | 6.434 | 233.54 | 43.03636 | 80.5 | In-house |
| 5 | 2-Methylbutyraldehyde | 6.732 | 244.7 | 41.06429 | 86.9 | In-house |
| 6 | 3-methylbutanal | 6.861 | 249.54 | 44.05 | 86.2 | In-house |
| 7 | 2-propanol | 6.926 | 251.96 | 45.05 | 79.3 | In-house |
| 8 | Ethanol | 7.307 | 266.21 | 45.05 | 85.4 | In-house |
| 9 | Valeraldehyde | 8.841 | 323.57 | 44.05 | 81.9 | In-house |
| 10 | 2,3-Butanedione | 8.879 | 325 | 43.05 | 86.1 | In-house |
| 11 | 2,4,7-Octanetrione | 9.028 | 330.58 | 43.065 | 84 | NIST 20s |
| 12 | isobutyl acetate | 9.978 | 366.1 | 43.05 | 82.6 | In-house |
| 13 | 2-methyl-3-buten-2-ol | 10.905 | 400.45 | 43.05 | 91.9 | In-house |
| 14 | 3-Hexanone | 11.157 | 405.82 | 71.1 | 89 | NIST 20s |
| 15 | Isoamyl Ether | 11.963 | 423.03 | 71.1 | 81.9 | In-house |
| 16 | 2-Methyl-1-propanol | 13.033 | 445.86 | 43.06667 | 94.3 | In-house |
| 17 | 2-Pentanol | 14.284 | 472.55 | 45.05455 | 98.4 | In-house |
| 18 | 3-methylbutyl acetate | 14.597 | 479.24 | 43.05 | 98.5 | In-house |
| 19 | 1-Butanol | 15.459 | 497.62 | 56.1 | 95.2 | In-house |
| 20 | Ethyl benzene | 15.58 | 500.2 | 91.07917 | 88.2 | In-house |
| 21 | 3-Carene | 16.386 | 517.39 | 93.1 | 81.6 | In-house |
| 22 | 2-heptanone | 17.434 | 539.77 | 43.05 | 92.1 | In-house |
| 23 | DL-2-Methyl-1-butanol | 18.323 | 558.74 | 57.1 | 90.9 | In-house |
| 24 | 3-Methyl-1-butanol | 18.352 | 559.34 | 55.0625 | 95 | In-house |
| 25 | cis-B-Ocimene | 19.764 | 589.49 | 93.09167 | 90 | In-house |
| 26 | Ethyl Hexanoate | 19.893 | 592.24 | 98.06667 | 82 | NIST 20s |
| 27 | 1-Pentanol | 20.392 | 602.78 | 42.05833 | 92 | In-house |
| 28 | 2-Methylpyrazine | 21.555 | 626.8 | 94.075 | 92.1 | In-house |
| 29 | Acetoin | 22.414 | 644.55 | 45.05 | 92.4 | In-house |
| 30 | Acetol | 23.3 | 662.85 | 43.04667 | 94.9 | In-house |
| 31 | 2-Heptanol | 23.551 | 668.02 | 45.05 | 87.9 | In-house |
| 32 | 2,5-dimethylpyrazine | 24.393 | 685.42 | 108.1 | 97.2 | In-house |
| 33 | 3-Methoxybutyl Acetate | 25.052 | 699.02 | 59.03333 | 76.7 | In-house |
| 34 | 2,6-Dimethylpyrazine | 25.274 | 703.61 | 108.1 | 86.6 | In-house |
| 35 | Ethyl Lactate | 25.78 | 714.05 | 45.05 | 84.5 | In-house |
| 36 | 3-Acetoxy-2-butanone | 26.94 | 738 | 43.04615 | 82 | In-house |
| 37 | 2-Nonanone | 27.035 | 739.97 | 58.05 | 86.3 | In-house |
| 38 | Nonanal | 27.272 | 744.86 | 57.06818 | 85 | In-house |
| 39 | 2,3,5-Trimethylpyrazine | 27.845 | 756.69 | 42.05 | 89.7 | In-house |
| 40 | Ethyl Octanoate | 28.947 | 779.46 | 88.05833 | 82.6 | In-house |
| 41 | Acetic acid | 29.954 | 800.26 | 43.05 | 78.5 | In-house |
| 42 | 2-Ethyl-3.5-dimethylpyrazine | 30.36 | 809.18 | 135.1 | 96.6 | In-house |
| 43 | Trans-Linalool oxide | 30.623 | 814.98 | 59.06 | 81.3 | In-house |
| 44 | Furfural | 30.926 | 821.63 | 95.05 | 79.1 | In-house |
| 45 | 2,3,5,6-Tetramethylpyrazine | 30.939 | 821.93 | 54.05 | 85.2 | In-house |
| 46 | 2-Prophyl-1-Pentanol | 31.297 | 829.79 | 57.1 | 87.5 | In-house |
| 47 | 2-Acetylfuran | 32.778 | 862.37 | 95.01667 | 72.1 | In-house |
| 48 | Benzaldehyde | 33.531 | 878.92 | 106.05 | 83.8 | In-house |
| 49 | 2,3-Butanediol | 33.537 | 879.05 | 50.05 | 98 | NIST 20s |
| 50 | Propanoic acid | 33.618 | 880.83 | 45.075 | 91 | NIST 20s |
| 51 | Linalool | 33.783 | 884.47 | 71.0625 | 74.4 | In-house |
| 52 | Trans-2-Nonenal | 34.208 | 893.81 | 56.07273 | 81.1 | In-house |
| 53 | Ethyl acetate | 34.604 | 902.53 | 43.05 | 85 | NIST 20s |
| 54 | Isobutyric Acid | 34.836 | 907.62 | 43.1 | 73.9 | In-house |
| 55 | Pivalic Acid | 35.15 | 914.52 | 57.1 | 70.4 | In-house |
| 56 | 1-Propanol | 35.719 | 927.04 | 43.05 | 81 | NIST 20s |
| 57 | Isoamyl Lactate | 36.354 | 940.99 | 43.07222 | 81.5 | In-house |
| 58 | Benzonitrile | 36.825 | 951.36 | 103.05 | 74 | In-house |
| 59 | Gamma-Butyrolactone | 38.131 | 980.07 | 42.06818 | 81.1 | In-house |
| 60 | Phenylacetaldehyde | 38.474 | 987.62 | 91.0625 | 71.9 | In-house |
| 61 | 2-Phenylpropionaldehyde | 38.792 | 994.6 | 105.05 | 72.5 | In-house |
| 62 | Butyric acid | 38.939 | 997.84 | 60.02 | 89.5 | In-house |
| 63 | Pentanoic acid | 38.962 | 998.36 | 29.05 | 88 | NIST 20s |
| 64 | Butanoic acid | 38.967 | 998.46 | 74.05 | 90 | NIST 20s |
| 65 | 4-Methyl-valeric acid | 45.6 | 1159.74 | 60.025 | 84.3 | In-house |

Remarks^: a^ (RT) Retention time in minutes

^b^ (RI) Retention Indices, calculated by standard FAEE (Fatty Acid Ethyl Esters, C4–C24)
